# Amiloidose por Transtirretina (ATTR) – Papel da Multimodalidade no Diagnóstico Definitivo

**DOI:** 10.36660/abc.20180077

**Published:** 2020-05-11

**Authors:** Tonnison de Oliveira Silva, Eduardo Sahade Darze, Luiz Eduardo Fonteles Ritt, André Luiz Cerqueira Almeida, Antônio Ximenes

**Affiliations:** 1 Hospital Cardio Pulmonar Centro de Estudos em Cardiologia SalvadorBA Brasil Hospital Cardio Pulmonar - Centro de Estudos em Cardiologia,Salvador, BA - Brasil; 2 Escola Bahiana de Medicina e Saúde Pública SalvadorBA Brasil Escola Bahiana de Medicina e Saúde Pública,Salvador, BA - Brasil; 3 Santa Casa de Misericórdia de Feira de Santana Feira de SantanaBA Brasil Santa Casa de Misericórdia de Feira de Santana,Feira de Santana, BA - Brasil

**Keywords:** Amiloidose/complicações, Pré-Albumina, Cardiomiopatia Restritiva, Fibrose Endomiocárdica, Hipertensão, Insuficiência Cardíaca, Diagnóstico por Imagem

## Introdução

A amiloidose por transtirretina (ATTR) é uma causa rara de cardiomiopatia restritiva e/ou polineuropatia periférica, de caráter progressivo, irreversível e fatal, subdiagnosticada e com diagnóstico definitivo realizado de forma tardia.^1^ O diagnóstico precoce, a caracterização do tipo de amiloidose e posterior instituição de terapêutica específica são fundamentais para uma evolução prognóstica mais favorável dessa doença.^1^ Apresentamos um caso de ATTR em que a suspeição clínica, associada ao uso da multimodalidade diagnóstica, notadamente da medicina nuclear, foram capazes de definir com segurança o diagnóstico, sem necessidade de biópsia.^2^

## Relato de Caso

**Figura 1:**
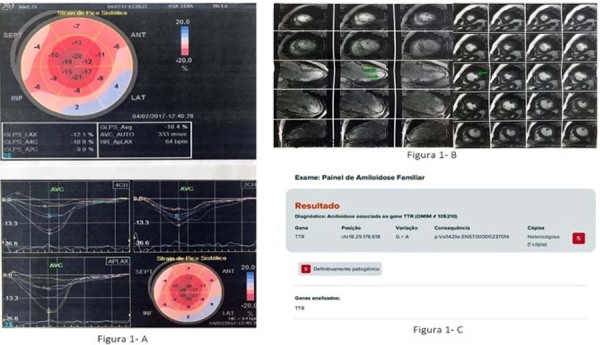
- 1-A) Ecocardiograma com strain demonstrando o clássico apical sparing .1-B) RM com realce tardio heterogêneo e difuso no ventrículo esquerdo (setas à esquerda) e evidenciando aumento difuso da espessura do VE (seta à direita).1-C) Resultado do estudo genético, demonstrando a mutação da valina pela isoleucina (VAL142IIe).

Paciente de 75 anos, do sexo masculino, com diagnóstico prévio de hipertensão arterial sistêmica (HAS) em estágio 1, com queixa de dispneia aos esforços moderados há dois meses. Fazia uso regular de losartana, 50mg ao dia, com controle pressórico adequado. Exame físico sem nenhum achado digno de nota, eletrocardiograma (ECG) mostrava ritmo sinusal de 64 bpm e extrassístoles ventriculares isoladas. Ecocardiografia (ECO) revelou discreta dilatação biatrial, discreta disfunção sistólica do ventrículo esquerdo, com fração de ejeção = 49% (Simpson), disfunção diastólica de grau II e presença de importante hipertrofia concêntrica do ventrículo esquerdo, desproporcional ao histórico de HAS, achados que levantaram suspeita de uma possível amiloidose cardíaca. Realizada ressonância magnética (RM) do coração ( Figura 1-B ), que demonstrou hipertrofia ventricular com realce tardio subendocárdico difuso e heterogêneo. ECO bidimensional pela técnica de *speckle tracking* evidenciou redução do *strain* global longitudinal (GLS = –10%), relação entre fração de ejeção e GLS = 4,9, com comprometimento difuso da deformação subendocárdica, porém com preservação do ápice ( Figura 1-A ), achados que reforçaram a suspeita clínica inicial.

**Figura 2:**
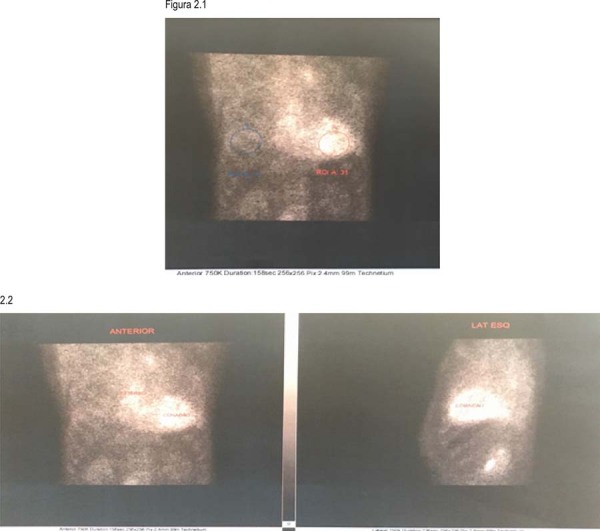
- Cintilografia miocárdica com pirofosfato marcado com tecnécio^99m^. 2.1) Relação de contagem entre o coração e a região correspondente no hemitórax direito = 1,8 (31/17 =1,8). 2.2) Concentração aumentada do radiotraçador em área de projeção do coração, em relação aos arcos costais, correspondendo ao escore 3. Escore >2 e relação de contagem entre o coração e a região contralateral > 1,5 indicam alta probabilidade de amiloidose transtirretina senil ou hereditária.

Solicitada imunofixação de proteínas no sangue e na urina, associada a pesquisa sanguínea de cadeias leves, todas negativas. Ainda sem diagnóstico conclusivo e diante da clínica e dos achados de imagem sugestivos, foi levantada suspeita de um tipo mais raro de amiloidose, a ATTR. Nesse tipo de amiloidose, os exames de laboratório não ajudam e as biópsias podem não ser conclusivas.^1 , 2^ Optou-se então pela solicitação de cintilografia com pirofosfato, de elevada acurácia diagnóstica para esse tipo de amiloidose.^2 , 3^ Os achados da cintilografia foram compatíveis com ATTR ( Figura 2 ).

O paciente foi submetido a estudo genético para diferenciação entre ATTRw(wild) e ATTRm (mutante) através de análise do DNA coletado em swab de saliva, que confirmou tratar-se de ATTRm, com mutação da valina por isoleucina ( Figura 1-C ). Apresentava também diagnóstico prévio de síndrome do túnel do carpo (STC) bilateral de causa não esclarecida; realizou-se uma eletroneuromiografia, que demonstrou polineuropatia axonal distal (característica de neuropatia amiloide).^1 , 5^ Iniciado tratamento com tafamidis fármaco que estabiliza a transtirretina, diminuindo a progressão da doença neurológica e que, mais recentemente, demonstrou benefício importante em termos de hospitalizações e mortalidade.^1 , 4^

## Discussão

A amiloidose é uma doença infiltrativa, localizada ou sistêmica, onde o grau de acometimento cardíaco pode definir o prognóstico. Trata-se de uma causa reconhecida de cardiomiopatia restritiva, insuficiência cardíaca (IC) e polineuropatia.^1^ Existem mais de vinte tipos de proteína amiloide, com maior destaque para duas: de cadeia leve (AL) e a relacionada a transtirretina (ATTR).^1^ No tipo AL, que é mais prevalente, mais comum em indivíduos idosos e do sexo masculino, as proteínas fibrilares são formadas por cadeias leves ( *kappa* e lambda) produzidas por plasmócitos dentro da medula óssea. A ATTRm, tipo mutante ou hereditária, é causada por uma mutação autossômica dominante e acomete de modo semelhante ambos os sexos, e o início do aparecimento dos sintomas se dá acima dos 60 anos; no entanto isso dependerá do tipo de mutação encontrado.^1^ Quanto à ATTRw, conhecida como tipo selvagem (“ *wild* ”) ou senil, não existe mutação associada e apresenta maior prevalência em homens > 70 anos.^1^ Os dois órgãos mais frequentemente acometidos pela amiloidose são o coração e o rim. Proteinúria importante, chegando a síndrome nefrótica e disfunção renal, são as principais manifestações do acometimento renal do acometimento renal dessa enfermidade clínica. A apresentação clínica de cardiomiopatia amiloide passa por um quadro de cardiomiopatia restritiva, IC direita, com ascite, hepatomegalia e edema dos membros inferiores, IC com fração de ejeção preservada e, menos frequentemente, um quadro similar ao de uma cardiomiopatia hipertrófica septal assimétrica.^1^ Comprometimento do sistema nervoso autônomo com hipotensão ortostática, do sistema nervoso periférico com polineuropatia sensitivomotora, desordens do sistema de condução e também STC, principalmente se bilateral, são algumas das possíveis manifestações de infiltração sistêmica pelo material amiloide.^1 , 5^ Em relação à STC, trabalho recente de Sperry et al.,^5^ demonstrou que parte dos pacientes com indicação cirúrgica tinha, na verdade, amiloidose como doença de base e, desses, 20% apresentavam também envolvimento cardíaco.^5^

A transtirretina é uma proteína sintetizada principalmente no fígado, com as funções de transporte de vitamina A e de tiroxina. Existem mais de cem mutações dos genes que codificam essa proteína levando em última análise, à formação de proteínas com dobramento errôneo e deposição extracelular dessas fibrilas amiloides nos nervos periféricos, autônomos e em órgãos como coração e rins.^1^ A AL e a ATTR apresentam diferenças no prognóstico e requerem estratégias terapêuticas completamente distintas.^3 , 5^ Desse modo, o diagnóstico precoce e a caracterização do seu tipo são cruciais para o manejo adequado dos pacientes.^2 , 3^

Por ocasião do raciocínio clínico, na presença de HVE ao ECO (principalmente diante de espessura septal >12mm) e de um ECG com baixa voltagem (BV), a hipótese diagnóstica de amiloidose cardíaca deve ser sempre aventada. No entanto, ressalte-se que esse achado clássico de dissociação entre ECG e ECO é muito pouco sensível (presente em 50% dos casos de AL e apenas 25% nos casos de ATTR).^1 , 6^ O ECO, nessa entidade clínica classicamente apresenta aumento da espessura ventricular, disfunção diastólica e, em estágios mais avançados, disfunção sistólica, porém são achados inespecíficos. Para que seja feito um diagnóstico com alta probabilidade de certeza, é necessária uma propedêutica mais avançada.^2 , 3 , 7 , 8^ A RM do coração e o ECO bidimensional com a técnica de *speckle tracking* apresentam boa acurácia, desempenhando papel importante no diagnóstico precoce dessa patologia.^6 - 8^ A RM, segundo o estudo de Austin et al.,^9^ apresenta, através da técnica de realce tardio, sensibilidade (S) de 88%, especificidade (E) de 95%, valor preditivo positivo (VPP) de 93% e valor preditivo negativo (VPN) de 90%.^9^ O acometimento é subendocárdico, podendo ser difuso, heterogêno ou transmural – esse último apresenta pior prognóstico.^8^ O *strain* cardíaco pode ser usado para diagnóstico diferencial de causas de aumento da espessura ventricular, com acurácia diagnóstica do achado da preservação do ápice bastante satisfatória (S = 96%, E = 88%, em pacientes sem doença arterial coronariana).^6 , 7^ Vale ressaltar que a presença do *apical sparing* não é exclusiva de doença amiloide podendo ser encontrada, por exemplo, em HAS, estenose aórtica, cardiomiopatia hipertrófica.^7^

No entanto, o achado de preservação do ápice, com RRSR ( *relative regional strain rate* , que representa a soma do *strain* apical ou basal + médio) > 1, associada a relação entre FE e GLS > 4,1 (como visto no caso em discussão), é altamente sugestivo de amiloidose.^6 , 7^ Tanto a RM quanto o ECO com *strain* podem sugerir adequadamente o diagnóstico de amiloidose cardíaca.^2 , 6 - 8^ A definição se trata-se de AL ou ATTR, informação fundamental para o cuidado desses pacientes, pode ser feita acuradamente por meio de medicina nuclear.^3 , 10^ A realização de cintilografia com tecnécio marcado com pirofosfato pode, na maioria das vezes, diferenciar esses tipos.^3 , 10^ Captação do radiotraçador em grau 2 ou 3 do escore de Perugini (avaliação visual) tem sensibilidade e especificidade em torno de 88%,com área abaixo da curva ROC de 0,945 (95%; IC, 0,901 a 0,977).^10^ Já a avaliação quantitativa, feita através da relação entre o coração e a região contralateral do tórax, ganha em acurácia, uma vez que um valor > 1,5 apresenta S e E em torno de 92%, com área abaixo da curva ROC de 0,960 (95%; IC, 0910 a 0,981).^10^ A maior casuística nesse cenário foi publicada por Gilmore et al.,^2^ com uma amostra de 1.217 pacientes sob suspeita de amiloidose, sendo que cerca de 360 pacientes tiveram confirmação diagnóstica feita através de cintilografia com pirofosfato, sem necessidade de realização de estudo histopatológico.^2^ Nesse estudo multicêntrico, nos pacientes sem gamopatia monoclonal, a medicina nuclear apresentou especificidade e VPP próximos de 100%.

**Figura 3:**
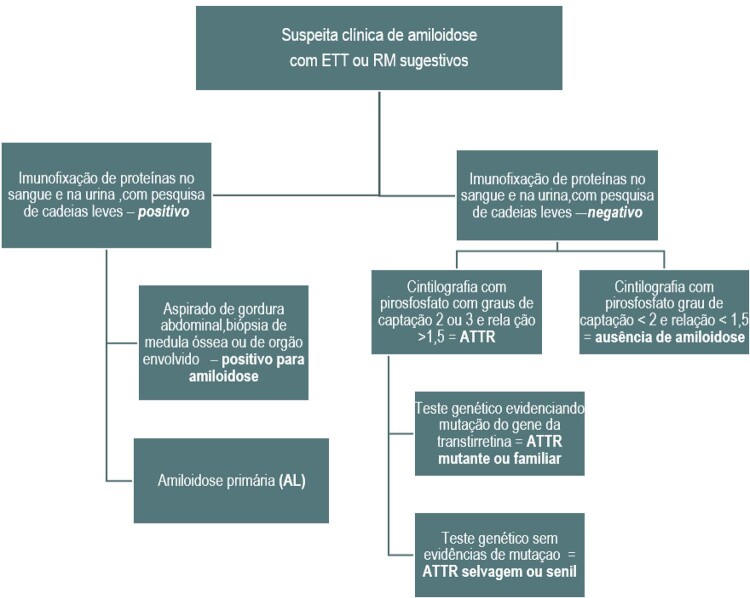
- Fluxograma simplificado para o diagnóstico de amiloidose.

Para os pacientes sob suspeita clínica e com ECO ou RM sugestivo da possibilidade de amiloidose, existe uma sequência diagnóstica a ser seguida.^2 , 3 , 10^ Inicia-se o fluxograma com a solicitação de imunofixação de proteínas no sangue e na urina, além da pesquisa de cadeias leves, em busca de amiloidose primária (AL). Para passar à outra etapa do algoritmo de investigação, é fundamental que esses exames laboratoriais iniciais sejam negativos; esse procedimento se explica pela existência de uma proporção de casos de AL com cintilografia positiva (podendo chegar a 27% de falsos positivos).^2^ Caso seja negativa a pesquisa de gamopatia monoclonal, a etapa seguinte é solicitação de cintilografia com Tc^99m^-pirofosfato, com o objetivo, dessa vez, de identificar depósitos de transtirretina no miocárdio^2 , 3 , 10^ ( Figura 3 ). Com a realização da multimodalidade, podemos identificar e diferenciar os tipos de amiloidose de forma precoce, com excelente acurácia e sem necessidade de biópsias.^2 , 3 , 6 - 8 , 10^

## Conclusão

Para pacientes sob suspeita clínica de amiloidose, na ausência de gamopatia monoclonal, deve-se prosseguir a investigação com a realização de cintilografia com pirofosfato, pois pode tratar-se de ATTR.^2 , 3 , 10^ A amiloidose, notadamente a associada a transtirretina, é uma doença cujo diagnóstico requer alto grau de suspeição clínica. É fundamental o diagnóstico precoce, pois trata-se de uma causa de polineuropatia e/ou cardiomiopatia que, se não for tratada, evolui de forma progressiva e letal.^1^
