# Supplementary material for: Amiloidose por Transtirretina (ATTR) – Papel da Multimodalidade no Diagnóstico Definitivo
Source: Arq Bras Cardiol. 2020 May 11;114(4 Suppl 1):8–12. [Article in Portuguese] doi: 10.36660/abc.20180077 (PMC8149115; doi:10.36660/abc.20180077)
Supplement: Translation

# Translation: Transthyretin Amyloidosis (ATTR) - The Role of Multimodality in the Definitive Diagnosis

**Authors:** Tonnison de Oliveira Silva, Eduardo Sahade Darze, Luiz Eduardo Fonteles Ritt, André Luiz Cerqueira Almeida, Antônio Ximenes

**Affiliations:** 1 Brazil Hospital Cardio Pulmonar - Centro de Estudos em Cardiologia, Salvador, BA - Brazil; 2 Brazil Escola Bahiana de Medicina e Saúde Pública, Salvador, BA - Brazil; 3 Brazil Santa Casa de Misericórdia de Feira de Santana, Feira de Santana, BA - Brazil

**Keywords:** Amyloidosis/complications, Prealbumin, Cardiomyopathy,Restrictive, Endomyocardial Fibrosis, Hypertension, Heart Failure, Diagnostic Imaging

## Introduction

Transthyretin amyloidosis (ATTR) is a rare cause of restrictive cardiomyopathy and/or peripheral polyneuropathy, of a progressive, irreversible and fatal nature, underdiagnosed and with its definitive diagnosis performed late.^1^ Early diagnosis, characterization of the type of amyloidosis and subsequent establishment of specific therapy are fundamental for a better prognosis of this disease.^1^ We present a case of ATTR where clinical suspicion associated with multimodality diagnosis — nuclear medicine — was able to safely deliver diagnosis, without the need for biopsy.^2^

## Case Report

Male patient, 75 years old, previously diagnosed with stage 1 systemic arterial hypertension (SAH), reporting dyspnea on moderate exertion for 2 months. He was regularly taking Losartan 50 mg a day, with proper blood pressure control. Physical examination with no relevant finding, electrocardiogram (ECG) showing sinus rhythm of 64 bpm and isolated ventricular extrasystoles. Echocardiography revealed mild biatrial dilation, mild left ventricular systolic dysfunction, ejection fraction = 49% (Simpson), grade II diastolic dysfunction and severe concentric hypertrophy of the left ventricle disproportionate to his history of SAH, which led to the suspicion of cardiac amyloidosis. Magnetic resonance imaging of the heart ( figure 1-B ) showed ventricular hypertrophy with diffuse heterogeneous subendocardial late enhancement. Two-dimensional speckle tracking echocardiography showed reduced global longitudinal strain (GLS = -10%), ejection fraction/GLS ratio = 4.9, with diffuse impairment of the subendocardial strain, but with preserved apex ( figure 1-A ), which reinforced the initial clinical suspicion.

**Figure 1:** - 1-A) Strain echocardiography showing classic apical sparing. 1-B) MRI with diffuse heterogeneous left ventricular late enhancement (arrows on the left) and diffuse increase in LV thickness (arrow on the right). 1-C) Result of genetic study showing valine to isoleucine mutation (VAL142IIe).

Immunofixation of proteins in blood and urine associated with blood search for light chains, all negative, was requested. Still without a conclusive diagnosis and in view of the clinical findings and suggestive imaging findings, a rarer type of amyloidosis, ATTR, was suspected. In this type of amyloidosis, laboratory tests do not help and biopsies may not be conclusive.^1 , 2^ Pyrophosphate scintigraphy was then requested because of its high diagnostic accuracy for this type of amyloidosis.^2 , 3^ The scintigraphy findings were compatible with ATTR ( Figure 2 ).

**Figure 2:** - Myocardial scintigraphy with technetium-99m labelled pyrophosphate. 2.1) Counting ratio between the heart and the corresponding site in the right hemithorax = 1.8 (31/17 = 1.8). 2.2) Increased concentration of the radiotracer in the projection area of the heart against the costal margin, corresponding to score 3. Score >2 and counting ratio between the heart and the contralateral region >1.5 have a high probability of senile or hereditary transthyretin amyloidosis.

The patient underwent genetic study to differentiate ATTRw (wild) x ATTRm (mutant), through DNA testing using saliva swab, confirming it is ATTRm, with valine to isoleucine mutation ( figure 1-C ). He had also been diagnosed with bilateral carpal tunnel syndrome with no clear cause. Electroneuromyography was performed and showed distal axonal polyneuropathy (characteristic of amyloid neuropathy).^1 , 5^ Treatment started with Tafamidis, a drug that stabilizes transthyretin, decreasing progression of neurological disease and, more recently, showing an important benefit in hospitalizations and mortality.^1 , 4^

## Discussion

Amyloidosis is a localized or systemic infiltrative disease, where the degree of cardiac involvement can define its prognosis. It is a recognized cause of restrictive cardiomyopathy, heart failure and polyneuropathy.^1^ There are more than twenty types of amyloid protein, most notably two: Light chain (AL) and transthyretin-related (ATTR).^1^ In the AL type, which is more prevalent, more common in the elderly and in males, fibrillar proteins are formed by light chains (Kappa and Lambda) produced by plasma cells in the bone marrow. Mutant or hereditary ATTRm is caused by an autosomal dominant mutation, similarly affecting both sexes, with the onset of symptoms above 60 years of age. However, this will depend on the type of mutation found.^1^ As for ATTRw, known as a “wild” or senile type, there is no associated mutation and it is more prevalent in men >70 years of age.^1^ The two organs most frequently affected by amyloidosis are the heart and the kidney. Severe proteinuria leading to nephrotic syndrome and renal dysfunction are the main manifestations of renal involvement of this clinical disorder. The clinical presentation of amyloid cardiomyopathy involves restrictive cardiomyopathy, right heart failure (HF), with ascites, hepatomegaly and lower limb edema, HF with preserved ejection fraction and, less frequently, a condition that is similar to that of an asymmetric hypertrophic septal cardiomyopathy.^1^ Impairment of the autonomic system with orthostatic hypotension, peripheral nervous system with sensory-motor polyneuropathy, conduction system disorders and also carpal tunnel syndrome (CTS), especially if bilateral, are some of the possible manifestations of systemic infiltration by amyloid material.^1 , 5^ Regarding CTS, a recent study by Sperry BW et al. showed that some of the patients with surgical indication actually had amyloidosis as the underlying disease and, of these, 20% also had cardiac involvement.^5^

Transthyretin is a protein synthesized mainly in the liver and carries vitamin A and thyroxine. There are more than one hundred mutations of the genes that encode this protein, ultimately leading to the formation of proteins with incorrect folding and extra-cellular deposition of these amyloid fibrils in the peripheral and autonomic nerves and in organs, such as the heart and kidneys.^1^ AL and ATTR present differences in prognosis and have completely different therapeutic strategies.^3 , 5^ Thus, early diagnosis and characterization of their type are crucial for the proper management of these patients.^2 , 3^

On clinical reasoning, the presence of LVH on the echocardiogram (especially if septal thickness >12 mm) and low voltage (LV) ECG, the diagnostic hypothesis of cardiac amyloidosis should always be considered. However, this classical finding of ECG X ECHO dissociation is very little sensitive (present in 50% of AL cases and only 25% in ATTR cases).^1 , 6^ The echocardiogram in this clinical entity classically presents increased ventricular thickness, diastolic dysfunction and, in more advanced stages, systolic dysfunction, but these are non-specific findings. In order to deliver a diagnosis with a high probability of certainty, a more advanced workup is necessary.^2 , 3 , 7 , 8^ Magnetic resonance imaging (MRI) of the heart and two-dimensional speckle tracking echocardiogram have good accuracy, playing an important role in the early diagnosis of this pathology.^6 - 8^ According to a study by Austin et al.,^9^ MRI with late enhancement presents 88% sensitivity (S) 95% specificity (E) 93% positive predictive value (PPV) and 90% negative predictive value (NPV).^9^ Impairment is subendocardial, and may be diffuse, heterogeneous or transmural, with the latter presenting the worst prognosis.^8^ Cardiac strain can be used for the differential diagnosis of causes of increased ventricular thickness, with diagnostic accuracy provided by the finding of quite satisfactory apex preservation (S = 96 %, E = 88%, in patients without coronary artery disease).^6 , 7^ It is noteworthy that the presence of apical sparing is not exclusive to amyloid disease, and can be found in SAH, aortic stenosis and hypertrophic cardiomyopathy, for example.^7^

However, the finding of apex preservation, with RRSR (relative regional strain rate, which represents the sum of apical/basal + medium strain) >1, associated with EF/GLS ratio >4.1 (as seen in this case), are highly suggestive of amyloidosis.^6 , 7^ Both MRI and strain echocardiogram can adequately suggest the diagnosis of cardiac amyloidosis.^2 , 6 - 8^ The definition of whether it is AL or ATTR, which is essential for managing these patients, can be done accurately using nuclear medicine.^3 , 10^ Scintigraphy with pyrophosphate-labeled technetium can differentiate, in most cases, these types.^3 , 10^ Uptake of Perugini grade 2 or 3 radiotracer (visual evaluation) have sensitivity and specificity around 88%, with an area under the ROC curve of 0.945 (95% CI, 0.901–0.977).^10^ Quantitative evaluation, done through the heart/contralateral chest area ratio, is best in terms of accuracy, since a value >1.5 presents S and E around 92%, with an area under the ROC curve of 0.960 (95% CI, 0910–0.981).^10^ The largest sample in this scenario was published by Gilmore et al.^2^ with a sample of 1,217 patients with suspected amyloidosis, where about 360 patients had diagnostic confirmation made through pyrophosphate scintigraphy not requiring histopathological study.^2^ In this multicenter study, in those patients without monoclonal gammopathy, nuclear medicine showed specificity and PPV close to 100%.

For patients with clinical suspicion, echocardiogram or MRI suggesting the possibility of amyloidosis, there is a diagnostic sequence to be followed.^2 , 3 , 10^ The flowchart begins with the request for immunofixation of proteins in the blood and urine in addition to assessment of light chains in the search for primary amyloidosis (AL). To move to the other stage of the investigation algorithm, it is essential that these initial laboratory tests be negative. This is due to the existence of a portion of cases of AL with positive scintigraphy (possibly reaching 27% false positives).^2^ If no monoclonal gammopathy is found, then the next step is to request 99mTc-pyrophosphate scintigraphy, for the purposes of, this time, identifying transthyretin deposits in the myocardium.^2 , 3 , 10^ ( Figure 3 ). With multimodality, we can identify and differentiate the types of amyloidosis early, with excellent accuracy and without the need for biopsies.^2 , 3 , 6 - 8 , 10^

**Figure 3:** - Simplified flowchart for the diagnosis of amyloidosis.

## Conclusion

Patients with clinical suspicion of amyloidosis, in the absence of monoclonal gammopathy, should continue the investigation with pyrophosphate scintigraphy, as it may be ATTR.^2 , 3 , 10^ Amyloidosis, notably that associated with transthyretin, is a disease that requires a high degree of clinical suspicion for diagnosis. Its early diagnosis is essential, as it is a cause of polyneuropathy and/or cardiomyopathy that, if left untreated, evolves progressively to death.^1^
